# Environmentally sustainable implementations of two-dimensional nanomaterials

**DOI:** 10.3389/fchem.2023.1132233

**Published:** 2023-03-03

**Authors:** Mehnaz Shams, Nikhita Mansukhani, Mark C. Hersam, Dermont Bouchard, Indranil Chowdhury

**Affiliations:** ^1^ Civil and Environmental Engineering, Washington State University, Pullman, WA, United States; ^2^ Departments of Materials Science and Engineering, Chemistry and Medicine, Northwestern University, Evanston, IL, United States; ^3^ National Exposure Research Laboratory, United States Environmental Protection Agency, Athens, GA, United States

**Keywords:** two dimensional nanomaterials, environmental implications, sustainability, graphene, emerging two dimensional nanomaterials

## Abstract

Rapid advancement in nanotechnology has led to the development of a myriad of useful nanomaterials that have novel characteristics resulting from their small size and engineered properties. In particular, two-dimensional (2D) materials have become a major focus in material science and chemistry research worldwide with substantial efforts centered on their synthesis, property characterization, and technological, and environmental applications. Environmental applications of these nanomaterials include but are not limited to adsorbents for wastewater and drinking water treatment, membranes for desalination, and coating materials for filtration. However, it is also important to address the environmental interactions and implications of these nanomaterials in order to develop strategies that minimize their environmental and public health risks. Towards this end, this review covers the most recent literature on the environmental implementations of emerging 2D nanomaterials, thereby providing insights into the future of this fast-evolving field including strategies for ensuring sustainable development of 2D nanomaterials.

## Graphical Abstract

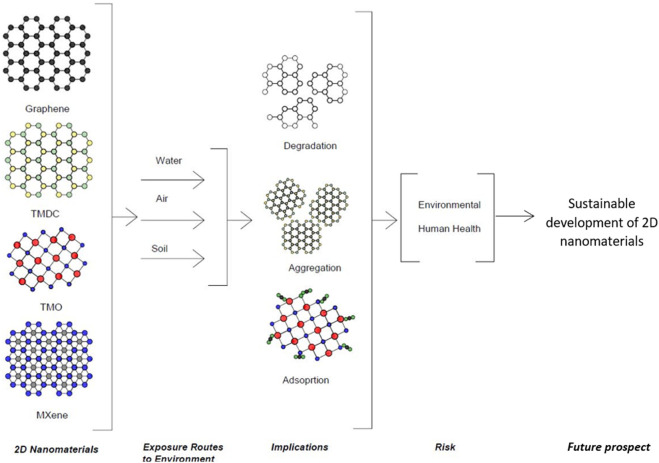

## 1 Introduction

Nanomaterials are defined as having at least one dimension of approximately 1–100 nm and are known for having unique and size-dependent optical, mechanical, electrical, and chemical properties. While relatively new, nanomaterials are entering the commercialization stage in many industries, including the electronic, magnetic, biomedical, pharmaceutical, cosmetic, energy, and paint industries, as well as for coatings and catalytic applications (Novoselov et al., 2012; Chung et al., 2013; Kemp et al., 2013). Two-dimensional (2D) nanomaterials are crystalline materials consisting of atomically-thin layers that possess strong ionic or covalent in-plane bonding while being stacked together by interlayer van der Waals bonding. There are several unique characteristics of 2D nanomaterials compared to their counterparts with different dimensionality and which makes them different from zero-dimensional (0D) nanoparticles, one-dimensional (1D) nanowires, and three-dimensional (3D) networks.

### 1.1 Why two-dimensional (2D) nanomaterials?

2D nanomaterials are of particular interest due to their exceptionally high specific surface area, making their surface properties dominant compared to their bulk counterparts. This high specific surface area makes 2D nanomaterials promising building blocks to construct functional composites as well as used as reinforced fillers to strengthen the resultant composites (Zhang, 2015). Moreover, these high aspect ratio sheet-like solids come in a wide array of chemical compositions, crystal phases, and physical forms, and are anticipated to enable a host of future technologies in areas that include electronics, sensors, coatings, barriers, energy storage and conversion, and biomedicine (Bianco et al., 2013; Kaul, 2014; Kalantar-zadeh et al., 2015; Cao et al., 2016).

With atomic-scale thicknesses, 2D nanomaterials possess maximum mechanical flexibility and optical transparency, making them promising for the fabrication of highly flexible and transparent electronic/optoelectronic devices (Geim and Novoselov, 2009). Moreover, the large lateral size and atomic thickness allow 2D nanomaterials to be highly favorable for many surface-active applications, such as electrocatalysis, photocatalysis, organic catalysis, and supercapacitors (An et al., 2016; Chen et al., 2019; Wu et al., 2019; Zhao et al., 2020a; Wang and Zhao, 2020; Chang et al., 2021; Ng et al., 2021).

Another attractive feature of 2D nanomaterials is that their electronic structures are highly sensitive to chemical modification, external electric fields, mechanical deformation, doping, and adsorption of other molecules or materials, which makes it easier to modify their electronic properties in a desired manner (Geim and Novoselov, 2009). Through chemical modification and integration into heterostructures, 2D nanomaterials are being integrated into a range of applications including highly conductive electrodes, planar spintronics, and high-efficiency catalysts (Yu et al., 2013; Kaul, 2014; Saadi et al., 2014).

2D nanomaterials have been extensively studied due to a vast array of unique physicochemical properties, such as high electronic conductivity, magnetic anisotropy, tunable band gap, and surface activity (Novoselov et al., 2004; Wang et al., 2012a; Chhowalla et al., 2013; Nicolosi et al., 2013; Li et al., 2014a; Xu et al., 2014). These properties arise from the quantum confinement of electrons.

The combination of excellent mechanical properties, light transmittance, and electronic properties makes 2D nanomaterials highly attractive in the fabrication of next-generation wearable, highly flexible, and transparent electronic/optoelectronic devices.

However, the synthesis, manufacturing, or application of these 2D nanomaterials can lead to unintended human exposures and environmental releases. These may pose a significant threat to public health and the environment. Even though the toxicity of 2D nanomaterials, their microbial degradation pathways, and their interactions with biological systems have been explored previously (Fojtů et al., 2017), for sustainable development of nanomaterials, it is important to have a better understanding of the fate and transport of these materials in the environment. The responsible development and applications of nanotechnology thus requires a coordinated and sustained research effort to understand and manage the environmental implications and human health risks of 2D nanomaterials.

In this review, literature on some of the emerging 2D nanomaterials (i.e., graphene oxide (GO), Molybdenum Disulfide (MoS_2_)) are summarized in terms of their environmental implications and a few prospects. By providing an overview of the properties and environmental implementations of 2D nanomaterials, rational strategies can be developed to help guide future sustainable development and safe best practices for the handling and utilization of 2D nanomaterials.

## 2 Graphene family nanomaterials

**FIGURE 1:**
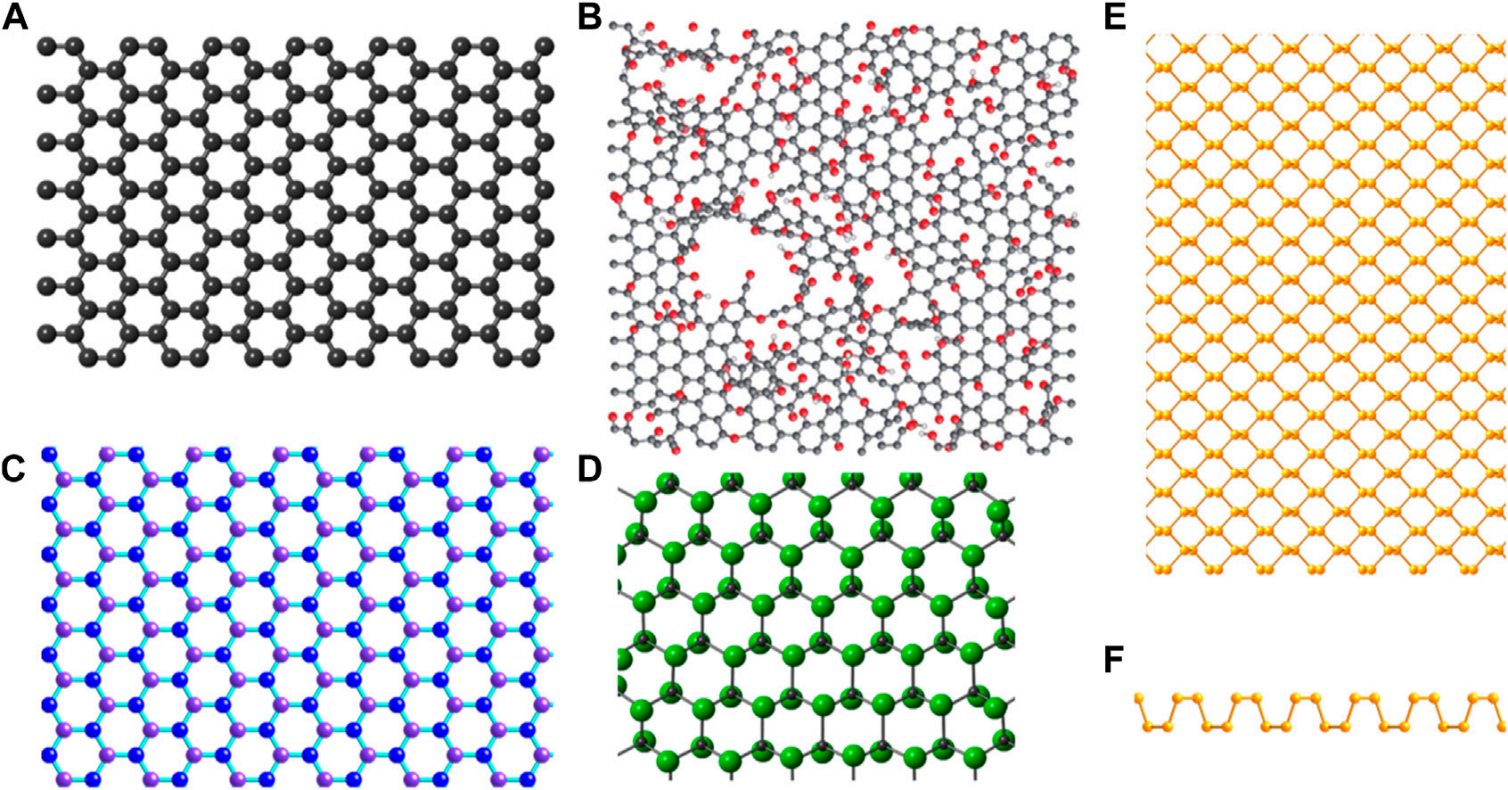
The structures of graphene family nanomaterials: **(A)** graphene, **(B)** graphene oxide, **(C)** hexagonal boron nitride, B shown in purple, N in blue, **(D)** fluorographene, F shown in green, C in gray. Fluorine atoms are distributed in one of two ways on the graphene surface in fluorographene, dubbed the “chair-type” and “boat-type conformations.” Depicted here is fluorographene in the more energetically favorable chair-type conformation. **(E)** Phosphorene top and **(F)** side views. **(B)** Reprinted with permission from Macmillan Publishers Ltd.: Nature Chemistry, **(A)**. Bagri, **(C)**. Mattevi, M. Acik, Y. J. Chabal, M. Chhowalla and V. **(B)**. Shenoy, Nat. Chem., 2010, 2, 581–587, Copyright 2010 (Bagri et al., 2010). All other structures produced by CrystalMaker9.

Graphene is recognized as the “mother of all graphitic forms,” i.e., the 2D building block of fullerenes, carbon nanotubes, and graphite, and has given rise to the wide range of GFNs studied today (Geim and Novoselov, 2007). Graphene nanomaterials vary in layer number, lateral dimension, surface chemistry, defect density, quality of the individual graphene sheets, composition, and purity. The properties and applications of some commonly used GFNs (Figure 1) have been summarized in Table 1 briefly.

**TABLE 1:** Properties and applications of graphene family nanomaterials.

Materials	Properties	Applications	Ref
Graphene• Monolayer of sp^2^ bonded carbon atoms in a honeycomb lattice.• Single layer graphene, few layer graphene (2–10 layers), and graphite nano- and micro-platelets	• Excellent mechanical property and thermal conductivity	• Composite materials, membranes, paints, and coatings	Frank et al. (2007), Balandin et al. (2008), Wang et al. (2010), Wassei and Kaner (2010), Zhang et al. (2010), Zhao et al. (2010), Liang et al. (2011), Zhang et al. (2011), Chang et al. (2012), Zhu et al. (2015), Dahanayaka et al. (2017), Kieu et al. (2017), Cataldi et al. (2018), Zhang et al. (2018)
	• Zero-gap semiconductor	• Solar cells, photocatalysts, sensors, and bioimaging agents	
		• Nanofiltration, membrane distillation, and pervaporation	
		• Electronics, motion and structural sensors, and reinforced bio-nanocomposites	
Graphene Oxide (GO)	• Carboxylate groups provide negative surface charge and colloidal stability in aqueous solutions	• Desalination by reverse osmosis, heavy metal removal, dye removal and adsorption	Loh et al. (2010), Dimiev et al. (2012), Huang et al. (2014a), Dervin et al. (2016)
Single-atom-thick carbon sheets with hydroxyl (-OH) and epoxide (-O-) functional groups on basal plane, and carboxylate (-COOH) groups on edges, introducing defects in lattice structure.	• Insulator	• Tissue engineering, removal of organic pollutants and antibacterial activity	
Reduced Graphene Oxide (rGO)	• Different reduction processes result in different properties, affecting the final performance of materials or devices	• For antibacterial coating and membrane	Fan et al. (2008), Wang et al. (2008), Williams et al. (2008), Zhou et al. (2009), Fernández-Merino et al. (2010), Mohanty et al. (2010), Shao et al. (2010), Pham et al. (2011), Feng et al. (2013), Shen et al. (2013), Chua and Pumera (2014), Li et al. (2016a), An et al. (2018)
Reduction of GO to reduce the functional groups and to heal the structural defects		• Sensing and energy storage applications	
Fluorographene	• Electrical and optical properties due to presence of fluorine	• Anti-corrosion and self-cleaning coatings	Balog et al. (2010), Robinson et al. (2010), Yin et al. (2018), Fan et al. (2020)
		• Desalination	
Two-dimensional carbon sheet of sp^3^ hybridized carbons, with each carbon atom bound to one fluorine		• Biosensor, electro-catalytic applications	
Hexagonal Boron Nitride (hBN) or white graphene	• Electrically insulating	• Thermal management material and lubricant in cosmetics, steels, paints, and sealants	Greim and Schwetz (2000), Elias et al. (2009), Zhi et al. (2009), Sainsbury et al. (2012), Cho et al. (2013), Jo et al. (2013), Gao et al. (2014), Hu et al. (2014), Tan et al. (2017)
Analogous to graphene in bulk structure. Each layer is composed of equal number of alternating B and N atoms in a honeycomb lattice	• Excellent thermal conductivity and mechanical properties, lubrication properties	• Hydrogen technologies such as fuel cells and water electrolysis	
	• Proton mobility, and chemical stability	• Antibacterial agent	
Graphitic carbon nitride (g-C_3_N_4_)	• Basic surface functionalities, electron-rich properties, H-bonding motifs etc.	• Effective water purification, water filtration and seawater desalination	Gillan, 2000; Yan et al. (2009), Liu et al. (2011a), Wang et al. (2011a), Su et al. (2012), Chu et al. (2013), Sano et al. (2013), Wang et al. (2013), Huang et al. (2014b), Kumar et al. (2014), Zhao et al. (2014), Ayán-Varela et al. (2015), Cao et al. (2015), Hu et al. (2017), Liu et al. (2018), Reddy et al. (2019)
Van der	• Stability, against heat and chemicals	• Oxidation of organic dyes and the inactivation of microorganisms	
Waals layered structure composed of solely carbon and nitrogen through sp^2^ hybridization	• Semiconducting properties	• Photocatalytic processes	
	• Insoluble in acidic, neutral, or basic solvents		

The table focusses mainly on the chemistry of the materials and some on the physical form of the materials.

### 2.1 Other graphene derivatives and elemental graphene analogues

Compared to the abundant literature on 2D materials like graphene and graphene oxide, the study of other graphene derivatives and elemental graphene analogues is still limited and at an early stage. Predictions and preliminary measurements of their properties confirm that they are complementary to conventional (that is, layered bulk-derived) 2D materials, which highlights that they deserve more attention as well in Tables 2, 3 (Zhang et al., 2017a; Mannix et al., 2017; Molle et al., 2017; Pumera and Sofer, 2017).

**TABLE 2:** Properties and applications of graphene derivatives and elemental graphene analogues.

Materials	Properties	Application	Ref
Graphane	• Electrical and optical properties due to the presence of hydrogen	• Hydrogen storage	Elias et al. (2009), Balog et al. (2010), Robinson et al. (2010)
Each carbon atom is sp^3^ bonded to a hydrogen atom	• Insulating properties	• Electronic device applications	
Graphyne and graphdiyne	• Extreme hardness, thermal resistance, conductivity or superconductivity, and through-sheet transport of ions	• Field emission, solar cells	Wan and Haley (2001), Marsden and Haley (2005), Haley, 2008; Du et al. (2011), Wang et al. (2012b), Yang et al. (2013), Li et al. (2014b)
Carbon hexagons bonded by linear acetylenic chains. (Figure 2)		• Photocatalytic activity	
Boron carbon nitride (BCN)	• Superior electrocatalytic activity	• Electrocatalysis and sensing	Wang et al. (2012c), Liu et al. (2015a)
Diamond-like structure combined with the sp Novoselov et al. (2012) σ-bonds among carbon, boron and nitrogen	• High electrical resistivity		
Black phosphorus (BP) or phosphorene	• Direct band-gap semiconductor	• High-performance electronic and optoelectronic device	Liu et al. (2014a), Wood et al. (2014)
Layered, phosphorus allotrope, held together by weak interlayer forces with significant van der Waals character. (Figure 1)	• High carrier mobility		
Silicene	• Dirac cone, high Fermi velocity, and high carrier mobility	• Quantum sensing, and energy devices	Salomon and Kahn (2008), Vogt et al. (2012), Zhao et al. (2016), Molle et al. (2018)
Low-buckled geometry with partial *sp* ^3^ hybridization and composed of group-IV elements	• Tunable band gap, and low thermal conductivity	• Adsorption of organic molecule	
Borophene	• Enhanced tunability, novel thermal and electronic properties, atomically thin and light	• Optically transparent electrode	Tang and Ismail-Beigi (2007), Yang et al. (2008), Wu et al. (2012a), Penev et al. (2012), Liu et al. (2013), Mannix et al. (2015), Adamska et al. (2018)
Triangular honeycomb lattice with a variable network of hollow hexagons (HHs) and characterized by anisotropy and polymorphism	• Energetically unstable due to three valence electrons	• Conductor or transistor	
Antimonene	• Band gap 2.28 eV	• Solar cells, sensors	Wang et al. (2015a), Zhang et al. (2015a), Pizzi et al. (2016), Abellán et al. (2017), Zhang et al. (2017b), Song et al. (2017), Song et al. (2018)
Buckled honeycomb lattice composed of group-V elements	• Enhanced stability and high carrier mobility	• Photocatalytic hydrogen evolution, photocatalytic degradation of pollutant	
Germanene	• Exhibits quantum spin Hall effect (QSHE)	• Transistors, photodetectors, optical devices, catalysts, energy storage devices, solar cells, thermoelectric devices, sensors, biomedical materials, and spintronic devices	Zhao et al. (2020b), Liu et al. (2021), Garg and Thakur (2022), Xi et al. (2022), Zhao et al. (2022)
• 2D Si and Ge layers	• Doping facilitates high-temperature superconductivity		
• Monolayer hexagonal structure			

**TABLE 3:** Properties and applications of 2D materials beyond graphene.

Transition metal dichalcogenides (TMDs)					
• Single plane of metal atoms between two separate layers of chalcogen atoms. General formula of MX2, where M is transition metal element and X is chalcogen. Two possible crystal structures: trigonal prismatic coordination with hexagonal closed packing (2H) or octahedral coordination with trigonal symmetry (1T)					

MATERIALS	PROPERTIES		APPLICATION	Ref	
Molybdenum Disulfide (MoS_2_)	• Unparalleled in its lubricity, temperature resistance, and stability		• Solid lubricant	Lauritsen et al. (2004), Splendiani et al. (2010), Eda et al. (2011), Radisavljevic et al. (2011), Chou et al. (2013), Bang et al. (2014), Feng et al. (2014), Finn et al. (2014), Gan et al. (2014), Wu et al. (2014), Liu et al. (2015b), Clark et al. (2015), Leong et al. (2015), Parzinger et al. (2015), Dervin et al. (2016), Shastry et al. (2016)	
• Hexagonal structure with similarities to graphene (Figure 3A)	• Mechanical strength, stability, and layer-dependent optoelectronic properties		• Transistors, photodetectors, and batteries		
• Depending on the stacking order between the layers, it adopts different crystal structures	• Direct band-gap semiconductors that exhibit strong photoluminescence		• Imaging agent, and photothermal ablation agent		
			• Catalyst in hydrodesulfurization reaction pathways		
			• Water purification treatments		
Tungsten Disulfide (WS_2_)	• Superlubricity, ambipolar behavior and electronic properties		• Catalyst for hydrogen evolution reactions	Feng et al. (2007), Lalwani et al. (2013), Notley (2013), Quinn et al. (2013), Voiry et al. (2013), Liu et al. (2014b), Cheng et al., 2014; Kaul (2014), Mahler et al. (2014), Sun et al. (2014), Dervin et al. (2016), Yue et al. (2018)	
Layered structure that adopts structure, like MoS_2_	• Direct band-gap semiconductors that exhibit strong photoluminescence		• Bone tissue engineering, nanoelectronic devices, water purification, and lithium-ion batteries		
Tungsten Diselenide (WSe_2_)	• Direct band-gap semiconductor		• Optoelectronic device	Tributsch (1978), Huang et al. (2014c), Zhang et al. (2014), Wang et al. (2016a)	
Layered semiconductor that shares its hexagonal crystal structure with MoS_2_ and WS_2_	• It has electron or hole charge carriers		• Heterostructure archetypes, such as MoS2-WSe2 alloys and WSe2 gold-plasmonic hybrid structures		
	• Enhanced photoluminescence				
Molybdenum Diselenide (MoSe_2_)	• Electrically tunable ambipolar behavior		• Laser technologies, catalyst for hydrogen evolution reactions	Pradhan et al. (2014), Saadi et al. (2014), Luo et al. (2015), Huang et al. (2016), Lei et al. (2016)	
Trilayers of molybdenum sandwiched between selenium ions causing a trigonal prismatic metal bonding coordination, but it is octahedral when the compound is exfoliated	• Higher electrical conductivity compared to MoS2		• Electrochemical biosensing of potent toxins		
**2D Transition Metal Carbides, Carbonitrides, and Nitrides (MXenes) (** Figure 3C **)**					
• General formula Mn+1AXn where M is a transition metal, X is carbon or nitrogen, and A is IIIA and IVA group elements and sometimes O, OH, or F. (n = 1, 2, or 3)		• High electrical conductivity and high hydophilicity	• Water purification, lithium-ion batteries, composites, and supercapacitors	Barsoum (2000), Naguib et al. (2011), Naguib et al. (2014), Yang et al. (2022)	
• Alternating MX and A layers joined with high-energy covalent/metallic/ionic character bonds		• Conductive or semiconductive			
**2D Oxides**					
Transition metal oxides (TMOs), such as TiO2, MoO3, and WO3		• Chemically stable, compatible with electrolytes, environmentally friendly compared to transition metal dichalcogenides	• UV-shielding and high dielectric properties		Osada and Sasaki (2009), Geng et al. (2010), Ma and Sasaki (2010), Geim and Grigorieva (2013), Wang and Sasaki (2014), Yuan et al. (2014)
Layers of corner-shared or edge-shared MO6, where M is the transition metal. (Figure 3D)		• Higher concentrations of vacancies	• Nanoelectronic and photochemical energy storage		
		• Magnetic and photoluminescent properties	• Catalysis and biomedical devices		

Researchers continue to isolate many new types of ultrathin 2D crystals, such as metal organic frameworks (MOFs), covalent organic frameworks (COFs), polymers, and ultra-thin metals (Huang et al., 2011a; Huang et al., 2011b; Colson et al., 2011; Duan et al., 2014; Kissel et al., 2014; Kory et al., 2014; Peng et al., 2014; Tan et al., 2014; Fan et al., 2015; Rodenas et al., 2015).

New classes of 2D materials and new polytypes within existing classes are continually being reported, greatly enriching the family of ultrathin 2D materials.

## 3 2D materials beyond graphene

Encouraged by the success and widespread applications of GFNs, researchers have explored other possible 2D structures beyond graphene and its derivatives. Studies with these materials have led to a vast library of 2D materials. (Geim and Grigorieva, 2013). Here, we introduce some of these categories and their relevant attributes.

**FIGURE 2:**
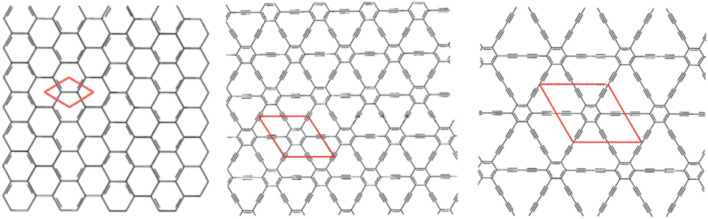
Structures of graphene (left), graphyne (middle), and graphdiyne (right). Each red parallelogram represents one unit cell. Reproduced by M. Inagaki and F. Y. Kang, J. Mater. Chem. A, 2014, 2, 13,193–13206, with permission of The Royal Society of Chemistry (Inagaki and Kang, 2014).

## 4 Environmental implications of graphene family nanomaterials

A broad and detailed understanding of the environmental implications of 2D materials will require knowledge of their release and transport through environmental media, distribution in environmental compartments, chemical and physical transformations, bioaccumulation, and effects on environmental organisms and ecosystems (Deng et al., 2011; Han et al., 2013; Chng et al., 2014; Wang et al., 2015b; Lanphere et al., 2015; Qian et al., 2015; Song et al., 2015). In the following sections, we survey previous work on the environmental implications of GFNs and 2D materials beyond graphene.

### 4.1 Environmental degradation of GFNs

With a burgeoning number of applications, the release of GFNs into the environment poses the risk of their transformation and degradation into other materials, such as carcinogenic polycyclic aromatic hydrocarbons (PAH) or comparatively benign carbon dioxide (CO_2_). This risk is particularly affected by their transport, which leads to a wider exposure risk. Thus, it is necessary to gather knowledge on the environmental behavior, fate, and transport of GFNs in the aquatic and terrestrial environments where many factors can influence their composition and behavior. It is especially important to assess their long-term impact in cases where oxidizing species may promote the disintegration of graphene into hazardous materials.

**FIGURE 3:**
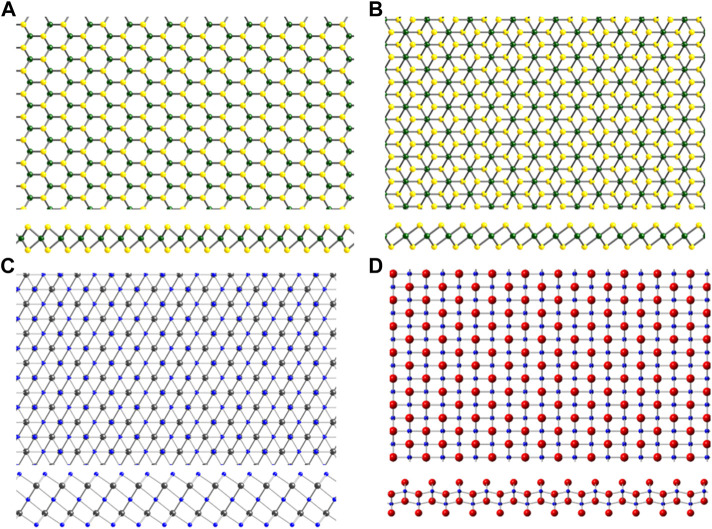
Structures of 2D materials that were discovered or have attracted renewed interest after the isolation of graphene. **(A)** Transition metal dichalcogenide (TMD) 2H crystal structure and **(B)** TMD 1T crystal structure. Here, transition metal atoms are shown in green, and chalcogen atoms shown in yellow. **(C)** Ti_3_C_2_ crystal structure representative of the MXene family. **(D)** 2D transition metal oxide Ti_0.91_O_2_^0.36-^. Ti shown in blue, C in grey, O in red.

#### 4.1.1 Sunlight-mediated transformations

**FIGURE 4:**
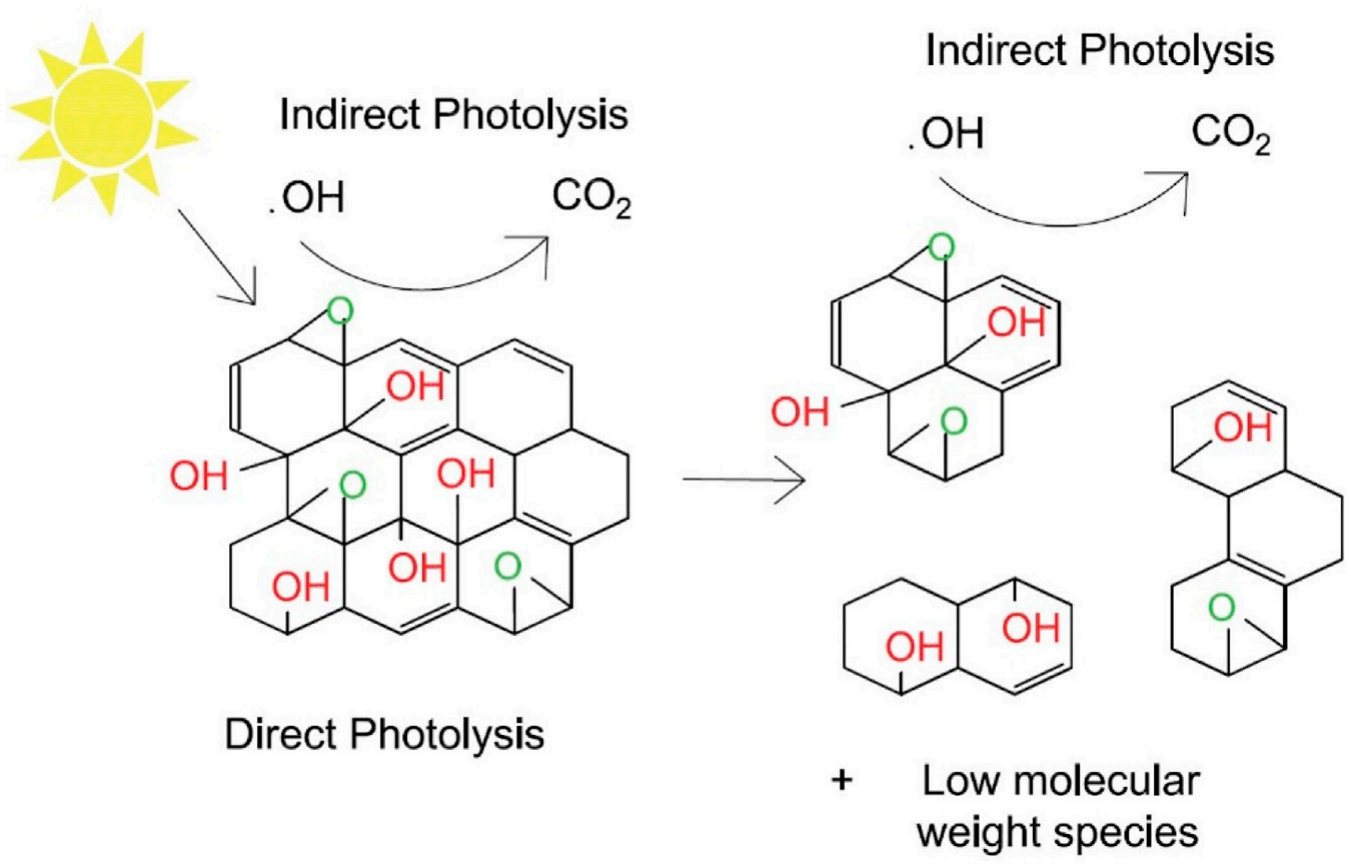
Pathways of direct and indirect photolysis of GO under sunlight. Reproduced with permission from W.-C. Hou, I. Chowdhury, D. G. Goodwin, W. M. Henderson, D. H. Fairbrother, D. Bouchard and R. G. Zepp, Environ. Sci. Technol., 2015, 49, 3435–3443. Copyright 2015, American Chemical Society (Hou et al., 2015). Reproduced with permission from Carbon, 110, Wen-Che Hou, W. Matthew Henderson, Indranil Chowdhury, David G.Goodwin, Xiaojun Chang, Sharon Martin, D. Howard Fairbrother, Dermont Bouchard, Richard G. Zepp, The contribution of indirect photolysis to the degradation of graphene oxide in sunlight, 426–437, Copyright 2016, with permission from Elsevier (Hou et al., 2016). Structures produced by AutoCAD 2017.

In some previous studies, it was demonstrated that under UV light irradiation, with or without Fenton reagent (Fe^2+^/Fe^3+^/H_2_O_2_), GO undergoes photoreduction, and CO_2_ forms due to photooxidation. These reactions are based on the photoreactions of oxygen-containing functional groups and carbon (Matsumoto et al., 2011; Koinuma et al., 2012; Zhou et al., 2012). Some other studies focusing on the chemical stability of the materials has shown that GO readily photo-reacts under simulated sunlight exposure, forming fragmented photoproducts similar to rGO as well as low molecular-weight species such as polycyclic aromatic hydrocarbons (PAHs) (Figure 4) (Zhou et al., 2012; Bai et al., 2014; Hou et al., 2015). When exposed to sunlight, graphene oxide degradation occurs mainly due to oxygen-containing functional groups on the basal plane through reduction and creation of holes (Shams et al., 2019).

Similarly, indirect phototransformation of GO presents another pathway of degradation in surface water (Lowry et al., 2012). Varied components of surface water, such as nitrates, minerals, and natural organic matter (NOM), can promote degradation by acting as chromophores and producing hydroxyl radicals, which are strong, non-specific oxidants that react with many nanomaterials in water.

However, the resulting byproducts from GO photodegradation can persist in water for a long period, and have different characteristics than their parent material (Hou et al., 2015). This makes the use of GO difficult especially where they will be susceptible to phtodegradation. Moreover, phototransformation will decrease the deposition rate of GO on many environmental surfaces such as those coated with Suwannee River Humic Acid (SRHA), which could be useful for the removal of GO from the environment. Another study has shown that rGO is less susceptible to photodegradation compared to GO (Shams et al., 2019). Hence, for coating and photocatalytic applications, use of rGO will be better compared to GO.

A study of the environmental instability of few-layer BP in ambient conditions suggests a photo-induced oxidation reaction of BP and degradation in the presence of oxygen absorbed in water (Favron et al., 2014; Ziletti et al., 2015). This degradation is a slower process, taking several hours to days but is dependent upon the thickness of the flakes. The degradation increases as thickness is reduced (Island et al., 2015).

However, these studies were performed in model condition in lab or with only with natural surface water. Whereas, these degradation rates, and by products can alter at different water condition. So more future research could be accomplished using different GFNs in other conditions such as in saline water. Also, oxidation of 2D materials in air, can have significant impact on the functional properties and the behavior of the materials which in result can also impact the degradation (Wang et al., 2017). Graphene usually has excellent oxidation resistance but at temperature higher than 250°C, or their structural defect graphene can play an important role in their oxidation (Liu et al., 2008; Barinov et al., 2009; Chen et al., 2011; Kang et al., 2012).

#### 4.1.2 Microbial transformations

There are some microbes (i.e., *E. coli*) that can degrade functionalized graphene compounds because graphene oxide acts as a terminal electron acceptor for heterotrophic and environmental bacteria (Salas et al., 2010; Akhavan and Ghaderi, 2012). Model environmental microbes from the genus Shewanella (a metal-reducing bacteria) also include a group of heterotrophic anaerobes that are found in lakes, oceans, marine sediments, and related environments (Hau and Gralnick, 2007). These microbes use different electron acceptors in their respiratory pathway to immobilize toxic metals and have environmental ubiquity, which makes them amenable to reactions with graphitic material. These reactions can further induce the biodegradation of GO, although they are dependent on some external factors. In addition, several enzymes like MPO and HRP can degrade graphene. However, the effectiveness of these enzymes rely on hydrophilicity, colloid stability and surface negative charge (Kurapati et al., 2015; Kurapati et al., 2017). Similar to bacteria one study found the use of fungi for graphene degradation with the help of LiP enzyme (Keli et al., 2018). This knowledge is useful for applications of environmental bacteria in green nanochemistries and for creating high performance nanomaterials (Salas et al., 2010; Wang et al., 2011b). Moreover, using oxidants for chemical degradation of graphene nanomaterials can be toxic to environment and costly. However, future research should also focus on the varied factors like temperature, presence of oxygen, pH etc. On the biodegradation of these nanomaterials. Moreover, the existing biodegradation studies only focused on GO and not on rGO, whose applications are also increasing with time. In addition, which specific enzyme is secreting from microbes and is responsible for the degradation should be studied in more detail.

#### 4.1.3 Disinfectant mediated transformations

Commonly used disinfectants in water distribution and treatment systems are chlorine, monochloramine, chlorine dioxide, ozone, and UV irradiation (Harza, 2005). In the United States, water purification and wastewater disinfection is accomplished almost solely by chlorination techniques. It was hypothesized that chlorine-based disinfectants in the water treatment environment significantly transform and degrade GFNs through oxidation, and that the resulting products, chlorinated GFNs and chlorinated PAHs, have increased mobility in the aquatic environment compared to the parent material (Frutos et al., 2011). Historically, halogenated PAHs are known to be toxic and carcinogenic (Fu et al., 1999). In some study, effect of photochlorination on GO was investigated (Li et al., 2016b; Du et al., 2017). These studies showed that photochlorination decomposes GO to rGO. The studies further showed that changes in oxygen containing functional groups of GO were due to the oxidation of the quinone groups in GO by chlorine, and further oxidation by Cl• and/or ClO• radicals. However, the mechanism of how the addition of functional groups to GFNs affects the toxicity or mobility of the degradation products remains unexplored. Also, how this change will affect aggregation, adsorption, transport, and interactions of GO with other surfaces needs to be investigated.

#### 4.1.4 Photocatalytic transformation

In one study, C_3_N_4_/graphene oxide (GO) aerogel was prepared to degrade methyl orange (MO), an organic contaminant, under visible light irradiation to 73% within 5 h in aqueous solution (Wan et al., 2016). In the study, contribution of C_3_N_4_/graphene oxide (GO) from adsorption and degradation was distinguished. This result was comparable to another study, where the composite was prepared similarly and MO degradation was noticed (Tong et al., 2015). In both the mentioned studies, the composite showed stable photocatalytic activity for MO degradation after four decomposition cycles. In another study, metal (Fe^2+^, Zn^2+^) was incorporated with g-C_3_N_4_ for rhodamine B (RhB) degradation. This study also showed that the composite can be regenerated and reused without appreciable loss of RhB degradation activity up to five cycles (Zhu et al., 2010). These results summarizes that g- C_3_N_4_ incorporated with other material has higher efficiency in pollutant degradation compared to pure g- C_3_N_4_ and shows excellent recyclability (Cheng et al., 2013; Li et al., 2013; Chen et al., 2014a; Zhang et al., 2015b). However, C_3_N_4_/GO aerogel has excellent adsorption ability, due to which, it is difficult to distinguish photocatalytic degradation from adsorption. While considering (RhB) degradation, there was no mention about the individual percentage of adsorption and degradation of the material (Zhu et al., 2014). Different synthesis approach of a composite, can result to the formation of a composite with different structure and distinctive properties. Overall it affects the surface area and catalytic activity of the composite (Zhu et al., 2005; Zhu et al., 2007; Li et al., 2014c). Effect of different synthesis techniques should be addressed in pollutant degradation and environmental remediation. Removal of g-C_3_N_4_ from the system after adsorption is barely mentioned in the above discussed studies. Even though, C_3_N_4_/GO aerogel can be easily separated by filtration from the reaction systems for recycling (Tong et al., 2015), other approach, like *in situ* methods for removal should be reviewed as well.

### 4.2 Toxicity of GFNs

Many nanoparticles can generate reactive oxygen species (ROS) due to their redox activity and cause oxidative stress to organisms. Among different nanoparticles, some researchers found that carbon nanotubes and graphene can penetrate plant cells and stimulate phytotoxicity at high doses (Lin and Xing, 2007; Liu et al., 2009; Stampoulis et al., 2009; Ghodake et al., 2010; Begum et al., 2011; Khodakovskaya et al., 2011; Anjum et al., 2013; Lee and Kim, 2014). The hydrophobic property and aggregation tendency of carbon based nanomaterials would enhance their capability to interact with many organic substances (De La Torre-Roche et al., 2013). Accumulation in addition to visible signs of necrotic damage lesions, all indicate an oxidative stress mechanism mediated through the necrotic pathway.

GO exposure can reduce swimming speed and cause settlement inhibition to aquatic organisms (Mesarič et al., 2013). Graphene can penetrate through the plasma membranes due to its sharp edges and cause cell death (Liu et al., 2011b; Begum and Fugetsu, 2013). Furthermore, graphene can significantly interact with cell membrane lipids due to its hydrophobic surface, and cause toxicity (Sanchez et al., 2012). This toxicity, may be due to the loss of membrane integrity, including initial cell deposition on graphene-based materials and membrane stress caused by direct contact with sharp nanosheets (Liu et al., 2011b). Besides concentration, toxicity also depends on the physicochemical properties of graphene, such as the density of the functional groups, size, conductivity, and chemical nature of the reducing agent used for deoxygenation of GO, as well as on the cell types exposed to the materials which needs to be explored further (Gurunathan et al., 2012). Similarly toxicity due to other graphene nanomaterials should also be assessed.

### 4.3 Aggregation and deposition of GFNs

**FIGURE 5:**
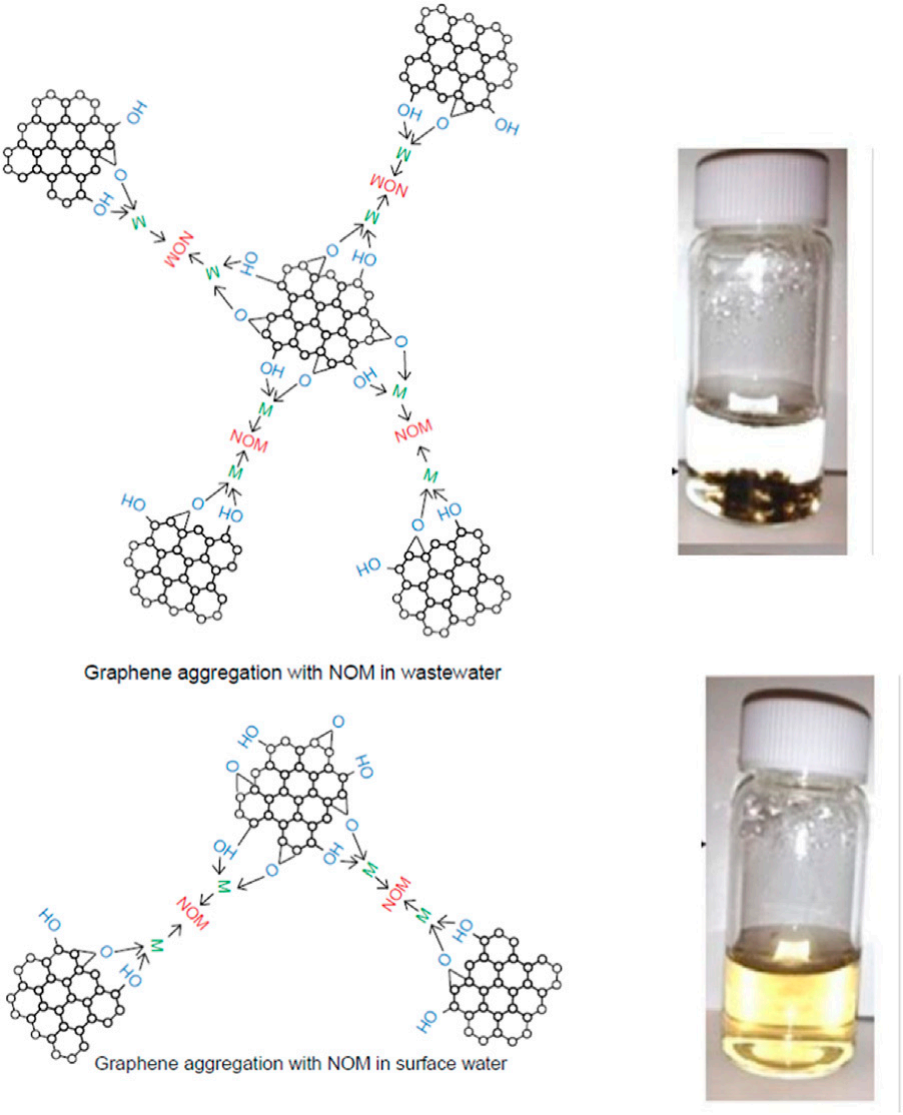
Aggregation & Stability of graphene oxide (due to presence of NOM and divalent cations (Ca^2+^, Mg^2+^) in surface water and wastewater. Reprinted with permission from I. Chowdhury, M. C. Duch, N. D. Mansukhani, M. C. Hersam and D. Bouchard, Environ. Sci. Technol., 2013, 47, 6288–6296. Copyright 2013, American Chemical Society (Chowdhury et al., 2013). Reprinted with permission from I. Chowdhury, N. D. Mansukhani, L. M. Guiney, M. C. Hersam and D. Bouchard, Environ. Sci. Technol., 2015, 49, 10,886–10893. Copyright 2015, American Chemical Society (Chowdhury et al., 2015b).

Aggregation and deposition of GO are dependent on various cations present in the aquatic and soil environments as they affect the surface charges of GO (Bargar et al., 1998; Ren et al., 2014; Duan et al., 2017). Recent studies indicate that GO can resist aggregation in natural and synthetic surface waters and can remain stable for extended periods due to steric repulsio. (Chowdhury et al., 2013; Wu et al., 2013). Figure 5 indicates that GO remains stable in natural surface water, but gets rapidly destabilized in effluent wastewater. Photo-transformed GO are significantly affected by the presence of CaCl_2_ with hydrodynamic diameter increasing with irradiation time, indicating an increased rate of aggregation (Chowdhury et al., 2015a). The deposition behavior also depends on many other factors, such as the presence of natural organic matter (NOM) (Chowdhury et al., 2015a). Presence of NOM and divalent cations (Ca^2+^, Mg^2+^) can bridge with GO functional groups, resulting in GO aggregates that settle from suspension (Chowdhury et al., 2015b). From this, it can be inferred that GO will sediment and may accumulate in biosolids and sludge during the wastewater treatment process. With successive reduction of functional groups, the colloidal stability of GO in water decreases (Chowdhury et al., 2015b; Shams et al., 2019). Deposition of photo-transformed GO on NOM-coated surfaces can reduce remobilization of GO in the aquatic environment (Chowdhury et al., 2015a).

### 4.4 Challenges in synthesis

Chemical vapor deposition, micromechanical exfoliation, epitaxial growth, and chemical reduction techniques are most widely used approach for synthesizing graphene (Gao et al., 2010). However, the existing synthesis approaches requires precise control over their compositions, thicknesses, lateral sizes, crystal phases, doping, defects, strains, vacancies, and surface properties to know the correlations between the structural features and properties. In chemical reduction technique, the use of reductant, usually hydrazine or dimethylhydrazine is highly toxic, which if inhaled by manufacturing workers, could cause serious health issues. The use of toxic reductant and other chemical stabilizers, to prevent aggregation, which are not biocompatible should be avoided. In a study, a “green” reduction technique of graphite oxide to graphene was showed using hydrothermal dehydration (Gao et al., 2010). Graphene of higher quality produced by liquid phase exfoliation of graphite, using solvents such as N,N-dimethylformamide (DMF), N-methyl-2-pyrrolidone etc. Should also be avoided as they are hazardous. Instead reducing sugars, such as glucose, fructose and sucrose could be used to synthesize graphene (Paton et al., 2014; Yi and Shen, 2014). Electrochemical methods to produce graphene also suffers from difficulty, in terms of cost and final product (Chen et al., 2014b; Parvez et al., 2014).

## 5 Environmental implications of 2D materials beyond graphene

Other 2D nanomaterials beyond GFNs are fast rising components in different industrial processes. Hence, these products have increasing potential to be released in the environment, thus necessitating studies of their environmental implications.

### 5.1 Transition metal dichalcogenides: Molybdenum disulfide

Among the range of 2D TMDs such as MoS_2_, WS_2_, MoSe_2_, and WSe_2_, the most research concerning environmental fate and dissolution processes has been conducted on MoS_2_ (Cheng et al., 2022; Liu et al., 2022; Liu et al., 2023). Hence, this section will also focus primarily on the environmental implications of MoS_2_.

#### 5.1.1 Sunlight mediated transformation

Many of the metal chalcogenides are stable under ambient conditions but can undergo environmental transformations (Chhowalla et al., 2013). Recent work on few-layer MoS_2_ shows dissolution over time upon exposure to environmental and biological simulant fluids (Wang et al., 2016b). These soluble products are formed due to photo-induced corrosion processes, where edge sites and defect sites are the primary degradation targets (Parzinger et al., 2015). However, the photodegradation rate of MoS_2_ has been observed to be slow under reduced oxygen concentration.

Metal phosphorus trichalcogenides can undergo photo-induced degradation or transformation in the environment, which sometimes provides interesting magnetic and ferroelectric properties as well as suitable band gaps for water splitting (Liu et al., 2014c). However, these can lead to the potential release of toxic ions such as Cu, Cd, Ni, or Co (Joy and Vasudevan, 1992; Evans and O’hare, 1994; Westreich et al., 2006; Dresselhaus, 2013; Ruiz-León et al., 2002; Venkataraman et al., 2003).

**FIGURE 6:**
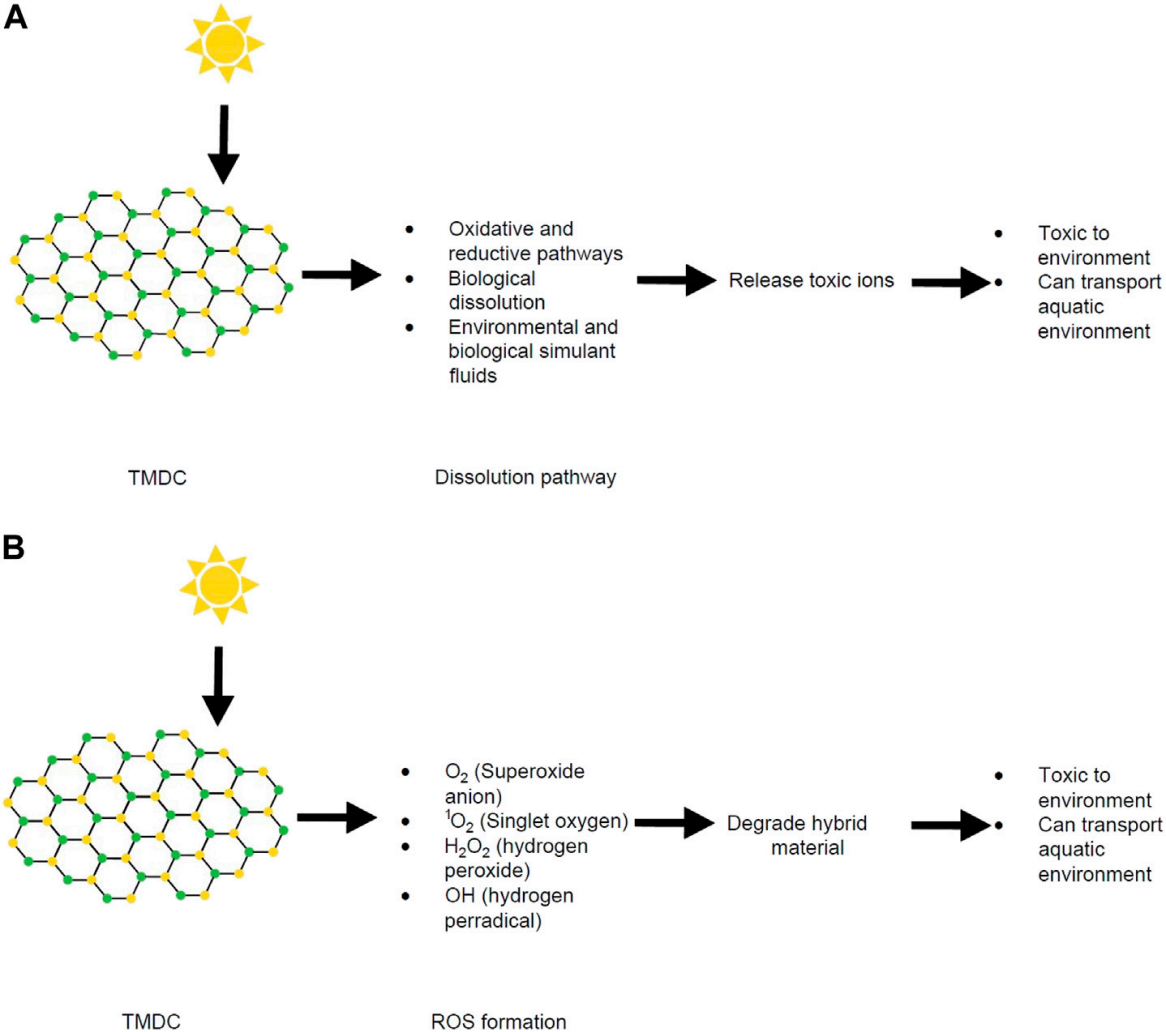
**(A)** Schematic diagram of dissolution process of TMDCs and their fate. **(B)** Schematic diagram of ROS formation from TMDCs and their fate. Structures produced by AutoCAD 2017.

Decreasing the size of MoS_2_ to only a few layers (−2–6 nm thick) increases the photocatalytic properties of MoS_2_ and ROS generation. These effects result from bandgap widening and the diffusion distance shortening for electrons and holes to the material surface. A previous study showed that four types of ROS (O_2_^•−^, ^1^O_2_, H_2_O_2_ and OH•) were present in few-layered vertically aligned MoS_2_ (FLV MoS_2_) (Liu et al., 2016). In the same paper, by decreasing the domain size, the bandgap of MoS_2_ was increased from 1.3 eV (bulk material) to 1.55 eV (few layer MoS_2_). This enabled the few layer MoS_2_ to generate ROS successfully (Liu et al., 2016). Similarly, hybrid materials made with MoS_2_ can have damaging effects due to the oxidative stress caused by ROS (Figure 6). For example, highly photocatalytically efficient MoS_2_/C_3_N_4_ (carbon nitride) heterostructures have a large potential for industrial applications due to their high quantum efficiencies and separation speed of electron−hole pairs (Li et al., 2014d). However, these heterostructures can be degraded by ROS and the resulting degradation products can have toxic effects in the environment. Specifically, multiple reports have explored the photodegradation of MoS_2_/C_3_N_4_ heterostructures (Pan et al., 2012; Hou et al., 2013a; Hou et al., 2013b; Ye et al., 2013).

However, since these studies were done with MoS_2_, more studies are required on other TMDs, and how other variables like structural defects, material thickness, oxidation time, temperature etc. Influence their degradation are required.

#### 5.1.2 Toxicity

In terms of toxicity, a study (Wu et al., 2016) showed the survival rate of *E. coli* in a dose dependent manner of molybdenum disulfide nanosheets. The results showed that high concentration (100 μg/mL) of molybdenum disulfide nanosheets, affects the metabolic profile of *E. coli* and the survival rate of *E. coli* was decreased. The mechanism was attributed to the fact that high concentrations of MoS_2_, caused damage to cell membranes, induced ROS accumulation, and reduced viability (Wu et al., 2016). On the contrary, another study shows that at similar concentration (100 μg/mL), few layer MoS_2_ nanosheets with small lateral dimension (<1 μm) did not induce any cytotoxic effect and cells maintained their viability (Shah et al., 2015). This observation is similar to other studies that have also showed that MoS_2_ and WS_2_ nanomaterials are non-cytotoxic (Teo et al., 2014). This implies that the fate of MoS_2_ in aquatic environments could be dependent on the type, lateral size, concentration, exposure time, number of layers, and chemical composition and surface functionalization of MoS_2_ (KenryLim, 2016).

#### 5.1.3 Challenges in synthesis

Currently there are some challenges in controlling the growth, overcoming the tendency toward aggregation and forming discrete nanosheets *versus* multi-pronged cores that lead to multi-site nanosheet growth of 2D nanosheet in TMDs (Terrones, 2016). Solution chemical synthesis can produce TMD materials in high yield and in solution-dispersible form, which also offers an increasingly interesting complement to traditional gas-phase, exfoliation, and substrate-bound synthetic platforms for accessing single- and few-layer TMD materials. Layered materials can also be exfoliated to monolayer and few-layer 2D nanosheets in various organic solvents *via* sonication but with low yield and not suitable for large scale production. In addition, the solvents used are expensive and toxic, and difficult to remove (Coleman et al., 2011).

### 5.2 Oxides

2D oxides have shown enormous potential in a broad range of application which necessitates the studies of their environmental implications. However, in terms of environmental implications, information on the 2D oxides is limited. For this reason, the review offers relevant information on bulk lamellar materials, which are often precursors for 2D materials, to give insight into fundamental chemistry.

#### 5.2.1 Toxicity

Similar to graphene nanoparticles, 2D-TiO_2_ nanoparticles could also produce reactive oxygen species upon interaction with organisms or ultraviolet radiation (Wang et al., 2007; Castiglione et al., 2011; Elghniji et al., 2012; Feizi et al., 2013; Paret et al., 2013). Oxygen free radicals formed during their photosynthesis process could accelerate the breakdown of organic compounds, cause quenching and increase the absorption of inorganic nutrients (Zheng et al., 2005; Yang et al., 2006). Furthermore, TiO_2_ nanoparticles tend to form a covalent bond with natural organic matter due to their small size, which results in larger surface area-to-mass ratio along with greater interaction with cells and gets transported to tissue and cells’ specific distribution (Castiglione et al., 2011; Huh and Kwon, 2011; Qiu et al., 2013; Song et al., 2013). However, it is considered that, the acute toxic effects of TiO_2_ nanoparticles do not follow a clear dose-effect relationship, due to their agglomeration and subsequent sedimentation.

On the contrary, TiO_2_ nanoparticles were observed to increase the plant growth by the improvement in nitrogen metabolism that promotes the adsorption of nitrate and photosynthetic rate (Yang et al., 2006; Gao et al., 2008; Wu et al., 2012b). Due to their antimicrobial properties, TiO_2_ could also increase a plants ability of absorbing and utilizing fertilizer and water, encouraging its antioxidant system, and hasten its germination and growth (Molina-Barahona et al., 2005).

TiO_2_ NPs shows potent toxicity to aquatic vertebrates (Bar-Ilan et al., 2013; Kim et al., 2014a; Kim et al., 2014b; Rosenfeldt et al., 2014). Even at ppb concentration, TiO_2_ NPs can generate (ROS) under solar irradiation, in a dose-dependent manner, which can accumulate in different organs and cause stunted growth, organ pathology, delayed metamorphosis and DNA damage (Kim et al., 2014a). In addition to dose, ROS generation is size dependent as smaller particles due to their large surface area can generate a higher level of ROS. From the study (Kim et al., 2014a), it can be concluded that TiO_2_ NPs mechanism of toxicity is mainly dependent on the surface area rather than its concentration. For organisms like E.coli, toxicity of TiO_2_ NPs mainly depended on the generation of ROS like OH radicals or oxidative stress in *E. coli* rather than the particle size and surface area (Pathakoti et al., 2013). However, the studies do not consider factors like flow, depth, temperature and presence of natural organic matter which can induce dissolution or aggregation of TiO_2_ NPs and make the condition of ecosystem more complex. Without careful application of these nanomaterials, they will eventually be present in the environment and may have long-lasting effects on aquatic life. Moreover, if 2D oxides l undergo biological dissolution, they may not persist in their original solid state, which could introduce new challenges (Goodman and Cheshire, 1982; Wang et al., 2016b).

#### 5.2.2 Environmental sensors

2D-MoO_3_ nanosheets has been extensively studied in gas and vapor sensing applications (Angiola et al., 2015; Ji et al., 2016). 2D-MoO_3_ is one of the most widely investigated gas sensitive materials, owing to its low cost, non-toxicity and stability at elevated temperature in air. The sensor using the 2D-MoO_3_ nanosheets has significantly a shorter response time as well as recovery time, compared to bulk MoO_3_ (Ji et al., 2016). However, synthesis technique of 2D-MoO_3_ nanosheets and fabrication technique of the sensor could cause aggregation, leading to a lower sensor response, which should be further investigated (Angiola et al., 2015). Also MoO_3_ is sensitive to environmental factors (humidity and oxygen), which has also not been considerd (Kamiya et al., 2004).

## 6 Gaps and future prospects

Complete materials characterization and mechanistic toxicity studies are essential for safe designing and manufacturing of 2D nanomaterials to develop applications with minimal risks for environmental health and safety. Moreover, future studies should focus on the effect of expanding concentration range of GO on these microorganisms and characterization of cell morphology for better comparison among studies.

For the development of next-generation membrane filtration systems for water purification, the primary challenge is to find the best combination of two-dimensional nanomaterials from GFNs and TMDs (e.g., MoS_2_ and WS_2_) that work together in membrane surfaces as antifouling and antibacterial agents. Findings from such studies will also apply to other areas including antifouling coatings for marine ship hulls, where fouling control remains a major challenge. Similar to graphene, 2D nanomaterials, such as TMDC, TMOs, metal-based nanocompounds, C_3_N_4_, BP, MXenes, hBN and other materials have also been researched for antibacterial applications (Mei et al., 2020). However, how the size, shape, layer numbers and surface functional modification, affects the antibacterial activities needs further research. In addition, majority of the research has been conducted on laboratories, with pure strain of a single microorganism (Yang et al., 2014; Wu et al., 2016; Kim et al., 2017). In the environment, there could be a mixed culture of microorganism, which could affect the antibacterial activity of these 2D nanomaterials and which should be looked as well.

Several challenges also exist for the efficient application of antimicrobial nanomaterials in drinking water treatment, such as the dispersion and retention of nanomaterials and the sustainability of antimicrobial activity. If nanomaterials are applied in the form of a slurry for water disinfection and microbial control, membrane filtration will be needed to retain and recycle the nanomaterials. Nanoparticles may also escape from the treatment system and enter the product water, which can have serious impacts on human health and ecosystems. Effective and reliable methods are needed to anchor the nanoparticles to reactor surfaces or to separate and retain suspended nanoparticles to reduce costs associated with material loss and to prevent human and environmental exposure. This includes developing better surface coating techniques, minimizing membrane fouling by nanomaterial suspension, and impregnating nanoparticles into filter packing materials, such as granular activated carbon or ion exchange resins.

Compared to graphene, which has been studied intensively, silicon- and germanium based 2D materials are much less explored, especially on their nanoscale level. This could be due to their synthesis, instability and a tendency toward oxidation. Moreover, the current knowledge about these materials covers only alkyl and aryl functional groups, and no other functionalities, whereas introduction of more complex functionalities may tune their physical properties similar to graphene (Hartman and Sofer, 2019). Constructing hybrid nanomaterials by using other 2D nanomaterials as building blocks, and thus further optimizing their properties and functionalities in future is a promising field.

There are many more 2D nanomaterials whose environmental implications, behavior, and fate are not yet known. It is essential to gather knowledge on their detailed material characteristics, toxicity, and implications so that preventive measures can be taken before the wholesale emergence of 2D nanomaterials in the market. In particular, it is important to relate specific physicochemical characteristics and functional assays so that predictions can be made for other materials and remediation can be designed accordingly. A challenge while utilizing these 2D nanomaterials is their high yield production to meet industry requirements for which more detailed research on their synthesis technique is required. Moreover, their preparation with desired structural characteristics in a highly controllable manner is still a challenge.

Although 2D nanomaterials have the potential to revolutionize aspects of electronics, medicine, and agriculture, the inherent risk of environmental and health hazards remain. In this regard, health and safety-focused research will augment application-driven research, ultimately enabling sustainable technological development.
